# Disrupted glutamate homeostasis as a target for glioma therapy

**DOI:** 10.1007/s43440-024-00644-y

**Published:** 2024-09-11

**Authors:** Mikołaj Biegański, Monika Szeliga

**Affiliations:** 1 Immunooncology Students’ Science Association, Medical University of Warsaw, Żwirki i Wigury 61, Warszawa, 02-091 Poland; 2 Department of Neurotoxicology, Mossakowski Medical Research Institute, Polish Academy of Sciences, Pawińskiego 5, Warszawa, 02-106 Poland

**Keywords:** Glutamate, Glioblastoma, Glutamate transport, Glutamate receptors, Neurotoxicity

## Abstract

Glutamate is the major excitatory neurotransmitter in the central nervous system (CNS). Gliomas, malignant brain tumors with a dismal prognosis, alter glutamate homeostasis in the brain, which is advantageous for their growth, survival, and invasion. Alterations in glutamate homeostasis result from its excessive production and release to the extracellular space. High glutamate concentration in the tumor microenvironment destroys healthy tissue surrounding the tumor, thus providing space for glioma cells to expand. Moreover, it confers neuron hyperexcitability, leading to epilepsy, a common symptom in glioma patients. This mini-review briefly describes the biochemistry of glutamate production and transport in gliomas as well as the activation of glutamate receptors. It also summarizes the current pre-clinical and clinical studies identifying pharmacotherapeutics targeting glutamate transporters and receptors emerging as potential therapeutic strategies for glioma.

## Introduction

Gliomas are a broad category of brain tumors divided into several distinct types each with its own spectrum of clinical manifestations, treatments, and outcomes [1]. Unlike most systemic tumors, gliomas have to deal with the constraints of the skull. In order to grow, these tumors have developed the ability to vacate space for expansion by destroying surrounding brain tissue. Twenty-five years ago, Sontheimer’s group demonstrated that glioma cells release large amounts of glutamate (Glu), which kills peritumoral neurons [2]. Over the following years, our understanding of the role of this neurotransmitter in the pathobiology of gliomas has increased significantly, laying the foundation for the development of new anti-glioma therapies. In this mini-review, we outline the role of Glu as a transmitter in the healthy brain. We also present the current knowledge on the involvement of Glu in shaping the malignant phenotype of gliomas and summarize preclinical studies and ongoing trials of inhibitors of Glu signaling.

### Overview of glioma

Gliomas represent a heterogeneous group of the central nervous system (CNS) tumors that arise from glial or precursor cells. They account for the vast majority of malignant CNS neoplasms. The most common gliomas are those of astrocytic origin, but there are also tumors of oligodendroglial or ependymal origin as well as mixed glioma [1]. The most prevalent symptoms include headaches, seizures, cognitive and motor deficits [3, 4]. Malignant gliomas are characterized by uncontrolled cell proliferation, infiltrative growth pattern, abnormal neovascularization, necrosis, resistance to apoptosis, and genomic instability [5–7]. Rapid cell proliferation results in the expansion of the tumor mass. Unlike systemic tumors, which have unlimited space for growth within soft tissue, gliomas can only expand within the skull. To overcome this spatial limitation and create space for expansion, these tumors destroy surrounding brain tissue. Gliomas have developed several mechanisms enabling adaptation to such a demanding environment. These mechanisms, contributing to the unfavorable clinical picture and glioma drug resistance, inextricably linked to disturbed Glu homeostasis, are discussed in the subsequent sections of this article.

Traditionally, the classification of these neoplasms has been primarily based on histology and immunohistochemistry, but the most recent classification has introduced several major changes that advance the role of molecular diagnostics in this regard [8]. The list of key diagnostic genes, molecules and pathways that are considered important for integrated CNS tumor classification is long, and its discussion is beyond the scope of this review. However, at least three of the earliest identified molecular markers should be outlined. Thus, co-deletions of the short arm of chromosome 1 (1p) and the long arm of chromosome 19 (19q), often detected in oligodendroglioma, predict a positive response to chemo- and radiotherapy in oligodendroglioma [9, 10]. Moreover, mutations affecting the genes encoding isocitrate dehydrogenase 1 or 2 (*IDH1* or *IDH2*, respectively) are associated with a longer overall survival of glioma patients [11]. These two aberrations have been incorporated into the definition of oligodendroglioma and astrocytoma in the current glioma classification. The third alteration linked with improved survival in glioma is hypermethylation of the promoter region of *MGMT*, a gene encoding a DNA repair enzyme, whose low level has been associated with increased sensitivity to the alkylating agents, such as temozolomide (TMZ), used in glioma therapy [12]. However, it should be emphasized that *MGMT* methylation status has not yet been incorporated into routine clinical practice either for prognostic evaluation or treatment decision-making due to the uncertainty of its predictive value and the lack of alternative treatment modalities [13]. Despite advances in understanding the pathobiology of gliomas, the prognosis for patients with these tumors remains poor. Depending on the report, median survival for glioblastoma, the most malignant and also the most common of gliomas, ranges from 8 [1] to 14 months [14]. The two main obstacles underlying treatment failure are molecular heterogeneity and phenotypic plasticity of glioblastoma cells. These two features make glioblastoma a dynamic and diverse ecosystem that interacts with the tumor microenvironment (TME). Particularly high degree of plasticity is displayed by glioma stem cells (GSCs), which significantly contribute to tumor heterogeneity [15, 16].

### Glutamate in the healthy brain

**Fig. 1:**
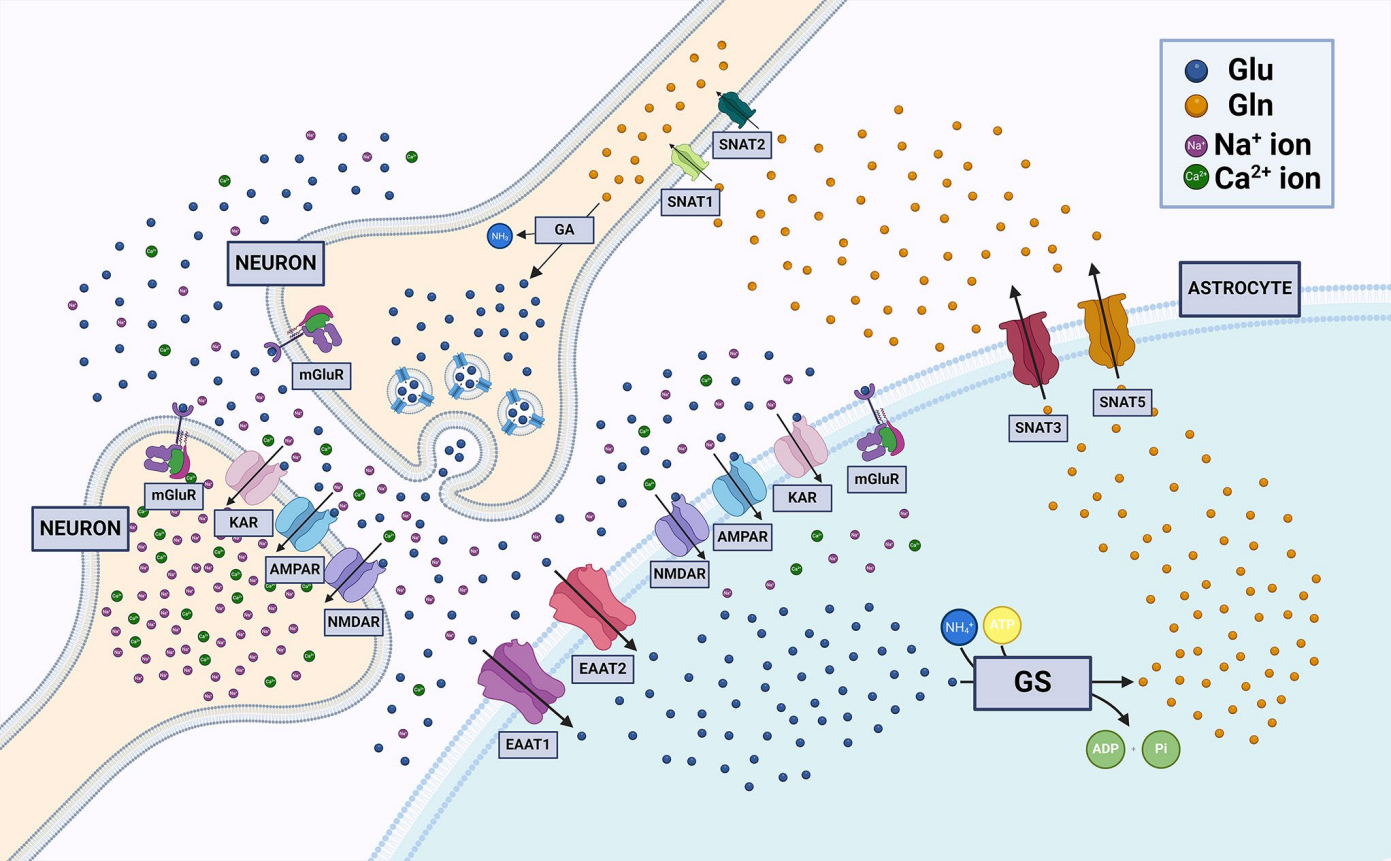
The Glu-Gln cycle. Glu is loaded into synaptic vesicles and released from activated neurons into the synaptic clefts. Once released, Glu binds to AMPA or NMDA receptors. Glu is removed from the extracellular space by EAAT1 and EAAT2. Taken up by astrocytes, Glu is amidated to Gln by GS. Eventually, Glu is transferred to neurons by transporters belonging to SNAT family. In neurons, Gln is reconverted to Glu by GA, thus closing the Glu-Gln cycle. AMPA, α-amino-3-hydroxy-5-methyl-4-isoxazolepropionic acid; EAAT, excitatory amino acid transporter; GA, glutaminase; Glu, glutamate; Gln, glutamine; GS, glutamine synthase; NMDA, N-methyl-d-aspartate; SNAT; sodium-coupled neutral amino acid transporter

Glu, the prevailing excitatory neurotransmitter, is synthesized in glutamatergic neurons from glutamine (Gln) by an enzyme glutaminase (GA, EC 3.5.1.2) [17]. Glu is loaded into synaptic vesicles by means of specific vesicular transporters, VGluT1 and VGluT2 [18, 19], and subsequently released from activated neurons into the synaptic clefts. Once released, Glu binds to either metabotropic glutamate receptors (mGluRs) or ionotropic glutamate receptors (iGluRs) to elicit distinct cellular responses on different time scales. Of note, there are four functional classes of iGluRs, namely α-amino-3-hydroxy-5-methyl-4-isoxazolepropionic acid (AMPA) receptors, N-methyl-d-aspartate (NMDA) receptors, kainate receptors and GluD receptors, the function of the latter as ion channels remains poorly understood [20]. To ensure the proper action of glutamatergic synapse transmission and to prevent excitotoxic neuronal damage, extracellular Glu concentration has to be maintained low [21]. To this end, Glu is removed from the synaptic clefts by transporters localized on the membrane of neurons and astrocytes. Human Glu transporters are termed excitatory amino acid transporters 1–5 (EAAT1–EAAT5) [22]. In rodents, glutamate/aspartate-transporter (GLAST) [23] corresponds to human EAAT1, and glutamate transporter 1 (GLT1) [24] corresponds to human EAAT2. These two proteins are abundant on astrocytic membranes and are the primary transporters involved in Glu clearance [22]. Although EAAT3 has been shown to be present in the majority of neurons throughout the CNS, its expression level in the adult brain is low, which is why this transporter does not play a main role in Glu removal from synapses [25]. Two other transporters, EAAT4 expressed exclusively in the cerebellar Purkinje cells, and EAAT5 detected in trace amounts in the brain, seem not to play a remarkable role in the transport of this amino acid [26]. Taken up by astrocytes, Glu is amidated to Gln by the astrocyte-specific enzyme glutamine synthase (GS, EC 6.3.1.2) [27]. Eventually, Glu is transferred to neurons by transporters belonging to the sodium-coupled neutral amino acid transporter (SNAT) family. Of these, SNAT3 and SNAT5 are mainly expressed in astrocytes [28, 29], while SNAT1 and SNAT2 are mainly neuron-specific [30, 31]. In neurons, Gln is reconverted to Glu by GA, thus closing the so-called Glu-Gln cycle, depicted in Fig. 1. In glioblastoma, virtually all of the elements of the Glu-Gln cycle described above are altered, which significantly contributes to the malignant phenotype of these tumors.

### Altered glutamate homeostasis in gliomas

#### Glutamate uptake

**Fig. 2:**
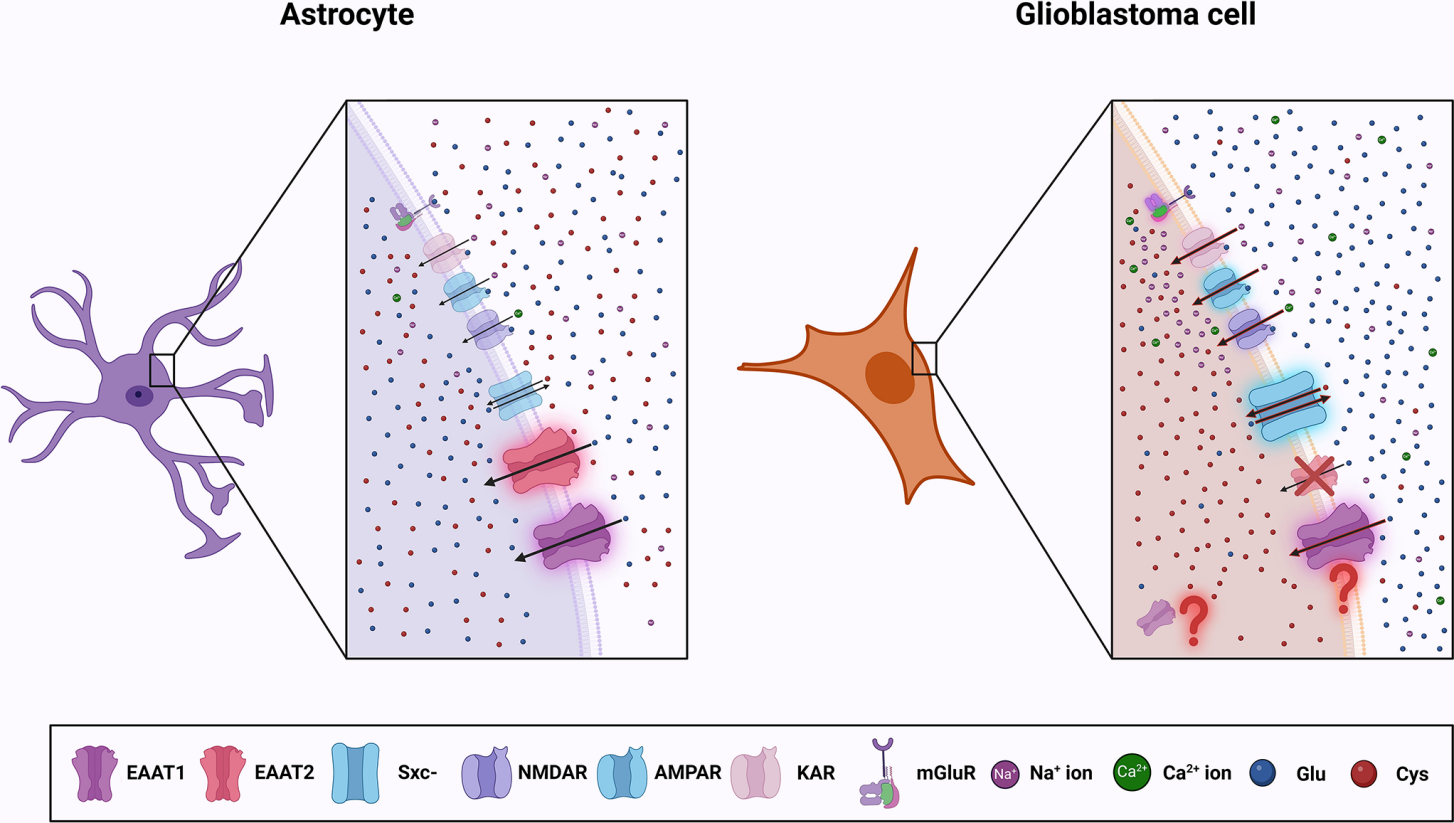
Glu transport is altered in glioblastoma. In the non-pathological brain, EAAT1, EAAT2 and Sxc^−^ cooperate to maintain Glu homeostasis. Glioblastoma cells release massive amounts of Glu due to high expression and activity of Sxc^-^ and aberrant activity of EAATs. EAAT2 is absent in glioblastoma cells, while the expression and localization of EAAT1 is unclear. EAAT, excitatory amino acid transporter; Glu, glutamate

Compared with normal rat astrocytes, human glioma cells present markedly reduced Na^+^-dependent Glu uptake [2], suggesting the aberrant expression of EAAT1 and/or EAAT2, the major Na^+^-dependent Glu transporters in glial cells. Indeed, Western blot analysis revealed only traces of EAAT2 in glioblastoma cells, and considerable amounts of this protein in astrocytes. Similarly, even though EAAT2 was detected in human brain tissues, it was almost undetectable in glioblastoma tissues [32]. Later studies showed that while EAAT2 is barely detectable in glioblastoma, it is present in less malignant gliomas [33, 34]. Its downregulation may result from an improper splicing of the transcript [35] or DNA methylation [36]. Additionally, an oncogene termed astrocyte elevated gene-1 (AEG-1), overexpressed in the vast majority of brain tumors, has been found to negatively regulate the expression of EAAT2 [37]. While the results obtained so far clearly document reduced expression of EAAT2 in glioblastoma, data on the function of EAAT1 in these tumors are less consistent. Western blot analysis showed prominent expression of EAAT1 in glioblastoma cells, but this transporter turned out to be localized in the intracellular cell fraction, not in the cell surface fraction. Nuclear localization of this transporter in both glioblastoma cells and biopsies revealed by immunofluorescence analysis [32] would suggest that EAAT1 cannot contribute to the Glu clearance from the extracellular space. A recent study showed no or low expression of EAAT1 in low-grade gliomas, and considerable amounts in most glioblastoma tissues examined. Noteworthy, EAAT1 was highly expressed on the plasma membrane of glioblastoma cells, and its level was inversely correlated with poor overall patient’s survival [38]. In the same study, both human and murine GSCs, which recapitulate the heterogeneity of tumors, presented significantly lower levels of intracellular Glu than normal astrocytes, while the level of extracellular Glu measured in the medium of GSCs was higher. Additionally, a marked reduction in extracellular Glu and an increase in intracellular Glu were achieved when EAAT1 was silenced with shRNA. These findings led the authors to hypothesize that EAAT1 may be involved in the release of Glu from the cell [38]. Further studies are necessary to elucidate the contribution of EAAT1 to Glu clearance in gliomas. Nevertheless, since both EAAT1 and EAAT2 play a crucial role in preventing the accumulation of toxic levels of Glu in the brain [39, 40], the alterations in their amount and localization translate into aberrations in the uptake of this amino acid (Fig. 2).

#### Glutamate release

In contrast to normal astrocytes, human glioblastoma cells not only exhibit remarkably lower rates of Glu uptake, but also release significant amounts of this amino acid. This phenomenon was first documented by Ye and Sontheimer, who showed that the level of extracellular Glu in the medium in which glioblastoma cells were cultured increased from 1 µM to above 100 µM within 5 h [2]. Later on, the release of such enormous amounts of Glu by glioblastoma cells was confirmed in several in vitro [41], ex vivo [42] and in vivo [43, 44] models using different measurement techniques. Remarkably, similar observation have been made in glioma patients [40, 41]. Thus, Marcus and colleagues utilized microdialysis to monitor the level of Glu in patients with malignant gliomas following surgery. The concentration of this amino acid was several hundred times higher in the tumor microdialysates than in peritumoral tissue samples [45]. Additionally, magnetic resonance spectroscopy revealed increased Glu levels in tumor tissues compared to contralateral normal brain tissues from glioblastoma patients [46], further corroborating that malignant gliomas release considerable quantities of Glu. This, in turn, raises the question of the mechanisms enabling Glu release by gliomas.

While in astrocytes Glu transport is primarily dependent on the presence of Na^+^, in glioblastoma cells more than 50% of Glu transport is Na^+^-independent and mediated by a carrier referred to as system x_c_^−^ (Sxc^−^) [2]. Sxc^−^ exchanges extracellular cystine (Cys) for intracellular Glu with a stoichiometry of 1:1 [47]. The uptake of Cys is a rate-limiting step in the biosynthesis of glutathione (γ-L-glutamyl-L-cysteinylglycine; GSH), an intracellular antioxidant responsible for reactive oxygen species (ROS) neutralization, critically important in the brain due to constant ROS generation in this tissue [48]. Sxc^−^ belongs to the family of heteromeric amino acid transporters (HATs), which are composed of a light subunit and a heavy subunit linked by a disulfide bridge [49]. The catalytic light subunit of Sxc^−^, termed xCT, confers transport and substrate specificity, whereas the heavy subunit, 4F2hc (also termed CD98hc), is necessary for trafficking of the heterodimer to the plasma membrane [49, 50]. Since 4F2hc is constitutively expressed on the membrane and may be coupled not only with xCT, but also with other partners to elicit different transporter activities [51], the expression of xCT is used as an indicator of Sxc^−^ activity.

Both subunits of Sxc^−^ are prominently expressed in human glioblastoma cell lines and patient tissues [41, 52, 53]. In some of the patient cohorts studied, increased xCT levels predicted shorter patient survival [52, 53] and were associated with seizures [53, 54] which are a common symptom in patients with gliomas [55]. A recent study documented an inverse correlation between the expression of *SLC7A11*, the gene encoding xCT subunit, and tumor suppressor p53 in glioblastoma tissues, and also provided strong evidence that p53 binds to *SLC7A11* and suppresses its expression [56]. Taking into account that p53 is often inactive in glioblastoma [57], it is tempting to speculate that the lack of active p53 underlies the high expression of *SLC7A11* in mutant p53-bearing tumors. Although xCT by itself does not seem to significantly influence the survival or proliferation of glioblastoma cells, its involvement in the secretion of Glu by these cells has been indisputably documented in experiments with siRNA [58] (Fig. 2).

#### Glutamate production

Gln is an important precursor for Glu synthesis in the brain and GA is considered the main producer enzyme of presynaptic Glu in the brain [59]. Glioblastoma cells display addiction to Gln, which in these cells becomes a conditionally essential amino acid [60]. Increased Gln uptake observed in glioblastoma cells is facilitated by the ASCT2 transporter, upregulated in these neoplasms [61, 62]. Genetic or chemical inhibition of ASCT2 decreases proliferation and migration of glioblastoma cells and their ability to form tumors in vivo thus extending the survival of glioblastoma bearing animals [62]. An upregulated transport of Gln results in its significantly increased levels in glioblastoma tissues compared with non-neoplastic brain tissue [46]. Once accumulated in the cell, Gln is intensively metabolized by GA encoded by the *GLS* gene. Notably, this gene, and particularly its isoform *GAC*, is overexpressed in glioblastoma [46, 63] while the other gene coding for GA, namely *GLS2*, is silenced in these tumors [63, 64]. Glutamine metabolism facilitates the synthesis of macromolecules and provides fuel for diverse metabolic pathways, thereby sustaining the survival and proliferation of cancer cells. Therefore, the silencing of *GLS* or the inhibition of GA activity suppresses glioblastoma cell survival and growth [65–68]. In line with these findings, GLS has emerged as an attractive target for glioblastoma therapy [60]. Regardless of the contribution of Gln per se in shaping the phenotype of gliomas, its intense metabolism translates into large amounts of Glu, which are released from the neoplastic cells due to the high activity of xCT.

### Disrupted glutamate homeostasis confers a biological advantage to gliomas

#### Glu-induced neuronal death provides space for growth

Elevated levels of extracellular Glu resulting from its impaired transport cause neuronal death, as first demonstrated in the experiments on glioblastoma-neuronal cocultures [2]. Regardless of whether neurons were in direct contact with glioblastoma cells, or were separated from them by a spacer, substantial neuronal death was observed after 40 h of culture. Neurotoxicity was almost completely prevented by the presence of either NMDA receptor antagonists, MK-801 and D(-)-2-amino-5-phosphonopentanoic acid (D-AP5), or mGluR antagonist, (S)-4-carboxyphenylglycine (S-4CPG). Notably, an AMPA receptor antagonist, cyanquixaline (CNQX), exhibited only partial neuroprotection, indicating that toxicity of glioblastoma cells was caused mainly by the activation of the NMDA or metabotropic Glu receptors [2]. The neurotoxic properties of Glu produced by glioma cells were confirmed in a subsequent study, in which subcloning of rat C6 glioma cells yielded clones with significantly different levels of Glu uptake/release ratios [43]. Thus, Glu-secreting glioma cells killed cocultured neurons, and treatment with MK-801 attenuated the neuronal loss. Furthermore, after implantation to rats, Glu-secreting cells formed tumors that expanded faster than tumors composed of wild type cells. Administration of MK-801 attenuated the growth of tumors formed by Glu-secreting cells [43], supporting the idea that the neurotoxic properties of glioblastoma cells are related to the release of Glu which activates NMDA receptors. It should be mentioned that regardless of the amount of Glu released, the cells displayed similar proliferative potential and ability to migrate [43]. Neither *xCT* silencing, nor treatment with 50 µM S-4-CPG, which inhibits xCT activity, altered glioma cell growth and proliferation, even though it significantly reduced Glu secretion. However, the application of conditioned media from *xCT*-silenced glioma cells to brain slices caused only sparse neuronal death, while conditioned media from wild-type cells induced massive neuronal degeneration. Both wild-type and xCT-depleted glioma cells implanted into organotypic brain slices formed tumors of comparable size, but neurodegeneration was much less pronounced in the *xCT*-knockdown group. Furthermore, compared with control animals, rats with *xCT*-silenced glioma cells implanted into brains presented a delayed onset and progression of neurological deficits, smaller perifocal edema, as well as prolonged survival [58]. Similarly, Robert and collaborators observed a marked reduction in the number of peritumoral neurons in mice bearing Sxc^−^-expressing gliomas compared to animals implanted with gliomas lacking Sxc^−^ [53]. Collectively, these observations indicate that glioblastoma cells have the innate ability to destroy surrounding neurons to create space for their growth.

#### Glu enhances cell migration and invasion

Glu released by glioblastoma cells activates AMPA receptors on the same cell and neighboring cells, which induces Ca^2+^ oscillations that are essential for cell migration. Of note, glioblastoma cells express AMPA receptors lacking GluR2 subunit, which are highly Ca^2+^-permeable [69, 70]. Conversion of these receptors into Ca^2+^-impermeable ones using the adenovirus-mediated transfer of the *GluR2* cDNA inhibited glioblastoma cell migration and invasion [69]. Treatment with GYKI or Joro spider toxin, inhibitors of AMPA receptors, completely abolished glutamate-induced Ca^2+^ oscillations and significantly reduced glioblastoma cell migration. In addition, cell invasion was compromised in vivo when Glu release was diminished by the administration xCT inhibitor, sulfasalazine (SAS), to mice bearing implanted human glioblastoma cells [41]. Further evidence for the role of AMPA receptors in glioblastoma migration was provided by Piao and collaborators, who showed that expression of GluR1, the most abundant AMPAR subunit in glioblastoma, correlates with increased cell-surface expression of β1 integrin and phosphorylation of focal adhesion kinase (FAK), the molecules implicated in glioblastoma invasion. Stimulation of the AMPA receptor leads to detachment of glioblastoma cells from the extracellular matrix (ECM). Moreover, overexpression of GluR1 facilitates invasion in a mouse xenograft model [71]. These results support the view that Glu release activates Ca^2+^-permeable AMPA receptors, which in turn enhances glioblastoma cell migration and invasion.

#### Glu stimulates cell proliferation and expansion of the tumor mass

Aside from enhancing cell motility, Glu also promotes proliferation. Mentioned above conversion of Ca^2+^-permeable AMPA receptors into Ca^2+^-impermeable ones, achieved by the overexpression of the GluR2 subunit, inhibited cell proliferation in vitro and reduced the rate of tumor growth in vivo [69]. Moreover, knockdown of GluR1 subunit decreased AMPA-mediated activation of mitogen-activated kinase (MAPK) and diminished cell proliferation in vitro as well as tumorigenicity in a xenograft model [72]. In line with these findings, chemical inhibition of AMPA with GYKI reduced glioma cell proliferation [73], although in a very recent study treatment with another antagonist of AMPA receptor, NBQX, showed no significant impact on this parameter [42]. It is worth mentioning that the same group documented a significant reduction in cell proliferation upon treatment with an antagonist of NMDA receptor, AP5. However, Glu appears to modulate proliferation not only through ionotropic receptors, but also through metabotropic receptors. Such an assumption can be made based on the observation that this parameter is reduced in glioblastoma cells treated with the mGlu2/3 receptor antagonist, LY341495, at the concentration of 1 µM, and this effect is partially reversed by co-treatment with the mGlu2/3 receptor agonist, LY379268. Of note, LY341495 treatment decreases the stimulation of the MAPK pathway [74], suggesting that the mGlu2/3-MAPK axis serves to support the proliferation of glioblastoma cells. These studies were extended by Arcella and colleagues, who documented diminished proliferation of LY341495-treated U87MG cells correlating with a decreased activation of the MAPK and phosphatidylinositol-3-kinase (PI3K) pathways. Furthermore, an administration of LY341495 inhibited the growth of U87MG implanted either subcutaneously or intracranially in mice [75]. Of note, intracranial models are superior in replicating the brain TME, therefore provide particularly valuable information. Interestingly, at the lower concentration of 60 nM LY379268 showed no effect on cell proliferation, but significantly potentiated the efficacy of TMZ [42]. When applied at the concentration of 100 nM, this compound reduced the proliferation of GSCs in vitro and their ability to form tumors in vivo [76], further indicating that mGlu3 receptors to some extent promote glioblastoma growth. Recent studies have demonstrated AMPA receptor-dependent functional synapses between neurons and glioma cells [77, 78]. Indeed, neuronal activity has been shown to promote glioma cell proliferation in vitro and tumor growth in vivo by secreting neuroligin-3, which activates the FAK and PI3K pathways [79, 80].

Downregulation of EAAT2 appears to be another mechanism used by glioma cells to enhance proliferation. Notably, its expression level inversely correlates with the grade of tumor malignancy, being the lowest in glioblastoma [33, 34]. Transfection with a vector carrying the *EAAT2* cDNA decreased proliferation and induced apoptosis in several human glioblastoma cell lines as well as inhibited growth of tumors formed by human U87MG glioblastoma cells subcutaneously in mice [33]. Delayed tumor growth and enhanced survival were also observed when C6 rat glioma cells genetically modified to express *EAAT2* were grafted in the striatum of rats [81]. Additionally, treatment with an agonist of the peroxisome proliferator activated receptor gamma (PPARγ), pioglitazone, increased the expression of *EAAT2* in human glioblastoma cells and reduced their viability, indirectly confirming the inverse correlation between the level of this transporter and cell survival [82].

Considering the role of EAAT1 in the biology of gliomas, it should be emphasized that both murine glioma GL261 cells and human GSCs expressing high levels of this transporter turned out to be significantly more aggressive in terms of survival compared to the low EAAT1-expressing cells after intracranial injection into mice. Moreover, when the cells expressing high amounts of EAAT1 were injected into mice, Glu concentration increased at both the injection site and in the contralateral hemisphere, and was higher than in tumors formed by the cells expressing low levels of EAAT1. Treatment with UCPH-101, a specific inhibitor of EAAT1, caused glioma cell death, but not astrocytes. Injected intratumorally, this compound decreased tumor mass and increased the survival of GL261 glioma-bearing mice, underscoring the involvement of EAAT1 in Glu trafficking in gliomas, and further confirming that this transporter supports glioma cell proliferation [38].

Finally, Sxc^−^ appears to be a molecule that contributes to glioma growth. Even though *xCT* silencing did not alter glioma cell growth and proliferation [58], pharmacological inhibition of this transporter with S-4-CPG or SAS significantly reduced the growth of glioblastoma cells in an L-cystine–dependent manner [58, 83]. Notably, both compounds reduced cystine uptake and intracellular GSH content in glioblastoma cells and the addition of cystine, but not Glu, resulted in a recovery of cell growth. Coincubation with a GSH analog replenished intracellular GSH levels and the growth of cells treated with either S-4-CPG or SAS. These results indicate that the status of GSH limits cell growth and both drugs affect this parameter by inhibiting cystine import for the synthesis of GSH [83]. GSH is an important endogenous antioxidant that neutralizes ROS, which in large quantities cause cell death. Glioblastoma cells generate high levels of ROS as a by-product of their metabolism. To survive, these cells enhance the activity of the antioxidant systems, an example of which is increased production of GSH [84]. Furthermore, the expansion of the tumor mass results in inability of blood vessels to oxygenate tumor cells. Decreased oxygen availability leads to hypoxia, which induces ROS production. Under hypoxic conditions, the dependence of glioma cells on GSH increases, therefore they augment surface expression of xCT, which leads to enhanced cystine uptake [85]. Additionally, elevated levels of antioxidants, including GSH, contribute to the development of resistance to ROS-generating chemo- and radiotherapy. Thus, since Sxc^−^ contributes indirectly to GSH production, it supports cellular resistance to treatment. This allows to speculate that inhibition of Sxc^−^ activity should sensitize glioma cells to ROS-inducing therapies. Indeed, knockdown of *SLC7A11* elevated ROS and decreased GSH levels [86], as well as enhanced invasive properties of glioblastoma cells [87], while its overexpression not only increases resistance to oxidative stress and TMZ [86]. The combined treatment with SAS and valproic acid, which has been proven to increase the level of ROS in glioma cells, turned out to potentiate cell death evoked by each of these drugs applied alone [88]. Furthermore, tumors expressing high Sxc^−^ showed a growth advantage compared to tumors expressing low Sxc^−^ and higher radiation resistance which was reduced by SAS [89]. In line with this finding, SAS potentiated the anti-tumor effect of gamma knife radiosurgery in rats with human glioblastoma xenografts, extending animal survival [90].

#### Glu contributes to the seizure activity

**Fig. 3:**
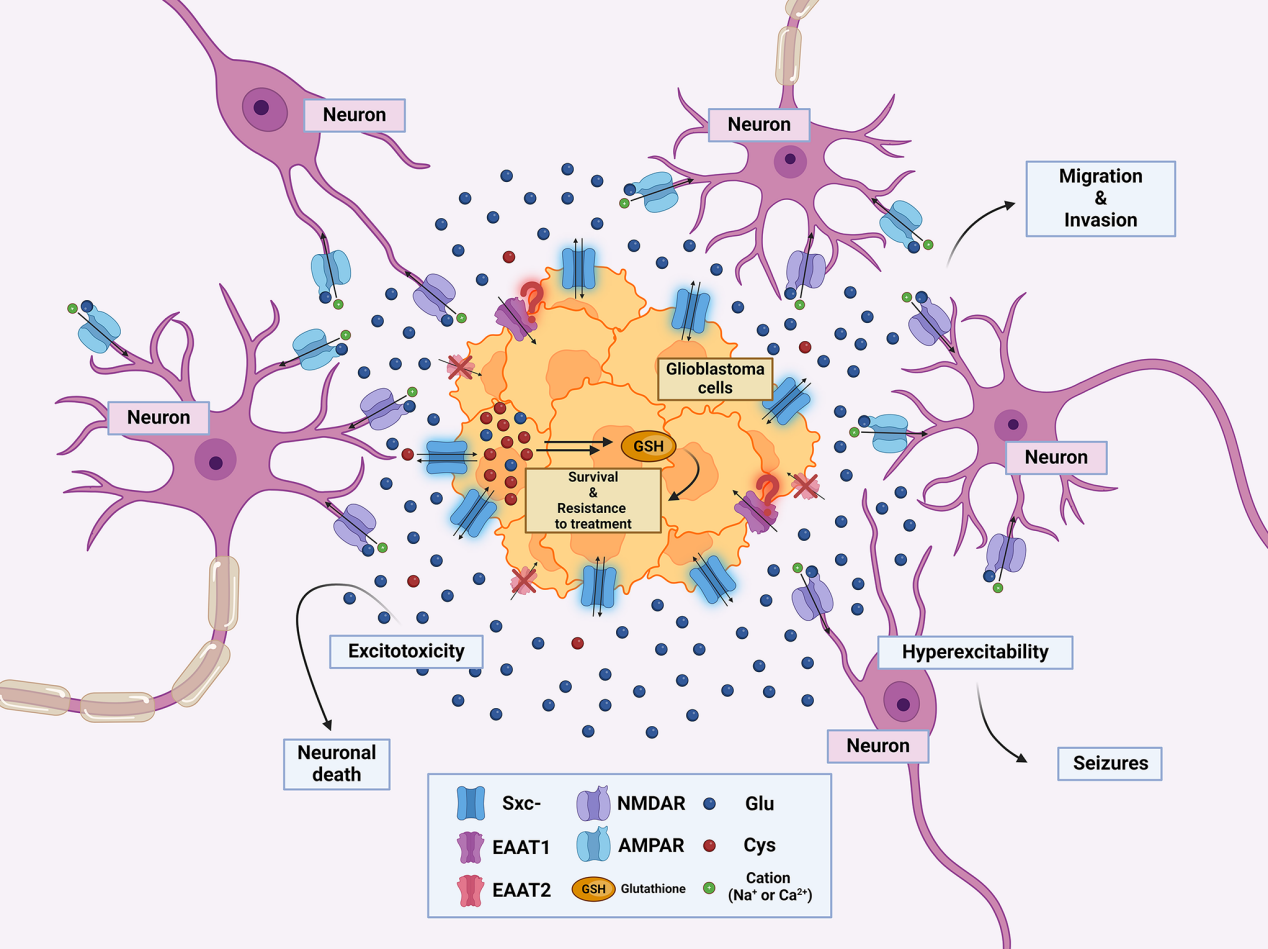
Disrupted Glu homeostasis confers a biological advantage to gliomas. Glu released by glioblastoma cells contributes to their proliferation, survival and resistance to treatment. It also supports migration and invasion of tumor cells. High Glu concentration in the tumor microenvironment destroys neurons surrounding the tumor, thus providing space for glioma cells to expand as well as confers neuron hyperexcitability, leading to epilepsy. Glu, glutamate

As already mentioned, seizures are a common symptom in patients with gliomas [55] and are nowadays known to be inextricably linked to Glu release from neoplastic cells. This connection was first documented by Buckingham and collaborators, who showed that glioma-bearing mice exhibit recurrent epileptic activity not found in control animals. Glu levels released from brain sections containing glioma are higher compared with sham sections and induce epileptic activity which is reduced by treatment with SAS [91]. This report was further extended in two studies from the same group showing that the tumor-associated brain is hyperexcitable and displays a persistent change in neuronal network activity [53, 92]. Altered glutamate homeostasis that confers a biological advantage to gliomas is depicted in Fig. 3.

### Glutamate signaling as a therapeutic target: from preclinical studies to clinical trials

The results presented above clearly document the involvement of Glu in shaping the malignant phenotype of gliomas. Glu neurotoxicity, detrimental to the brain tissue surrounding tumor, allows for the creation of space for growth, which translates into an extreme infiltrative capacity of these tumors. Moreover, Glu contributes to the growth and survival of gliomas, both through interaction with receptors and subsequent activation of signaling pathways, as well as by participating in the synthesis of GSH. This multifaceted role of Glu in glioma pathobiology supports the rationale for pharmacological targeting of Glu receptors and transporters. Some of the preclinical studies and the clinical trials of Glu signaling inhibitors are summarized below. Clinical trials are summarized in Table 1.

**Table 1:** Clinical trials of glu-signaling inhibitors

Drug(s)	Class	Outcome	Clinical trial no	Ref.
SAS	Sxc^−^ inhibitor	No clinical response	ISRCTN45828668	[82]
SAS + TMZ + radiation therapy	Sxc^−^ inhibitor	No change in OS and PFS prolonged SFS	-	[83]
SAS + stereotactic radiosurgery	Sxc^−^ inhibitor	No results published	NCT04205357	-
SAS + gabapentin + memantine + TMZ/radiotherapy	Sxc^−^ inhibitor Thrombospondin-1 inhibitor NMDAR antagonist	Ongoing trial Still recruiting (as of June 2024)	NCT05664464	[86]
Telampanel	AMPAR antagonist	Poor clinical response (1/19 patients)	NCT00064363	[93]
Telampanel + TMZ + radiotherapy	AMPAR antagonist	mOS increased from 14.6 to 20.3 months Drug well tolerated	NCT00267592	[94]
Perampanel	AMPAR antagonist	Ongoing trial	NCT03636958	-
Perampanel	AMPAR antagonist	Ongoing trial	EU-CT number: 2023-503938-52-00 30.11.2023.	[95]
Memantine	NMDAR antagonist	No results published	NCT01260467	-
Memantine	NMDAR antagonist	Ongoing trial	NCT01430351	-

AMPAR, α-amino-3-hydroxy-5-methyl-4-isoxazolepropionic acid receptor; Glu, glutamate; N-methyl-d-aspartate receptor; OS, overall survial; PFS, progression free survival; SAS, sulfasalazine; SFS, symptom free survival; Sxc^-^, system xc^-^; TMZ, temozolomide

### Targeting Glu transporters

In the preclinical studies presented above SAS, approved by the Food and Drug Administration (FDA) to treat inflammatory bowel disease, displayed a promising anti-glioma activity. Even though this drug has several weaknesses, such as a short half-life or susceptibility to cleavage by gut bacteria [93], so far it is the only approved drug penetrating the blood-brain barrier and targeting Sxc^−^. These premises encouraged the initiation of a double-blinded, prospective, phase 1/2 clinical trial (ISRCTN45828668) of SAS as a single agent for the treatment of recurrent or progressive high-grade gliomas. In the group of 10 patients, no objective clinical response to SAS was observed; on the contrary, rapid tumor growth despite SAS treatment and serious side effects occurred. The lack of therapeutic benefit led to the early termination of this trial [94]. In another trial, 12 patients with glioblastoma were treated with TMZ and SAS with radiation therapy after surgery. This treatment did not change either median overall survival (OS) or progression-free survival (PFS), but strongly, although not significantly prolonged seizure-free survival (SFS). The study was discontinued due to the high rate of side effects, especially hematologic toxic effects [95]. A more recent phase I clinical trial (NCT04205357) has been designed to evaluate the safety associated with the addition of SAS to stereotactic radiosurgery for recurrent glioblastoma. This study was completed in 2022, but still no results have been published. Another, still recruiting (as of June 2024) trial (NCT05664464) aims to evaluate whether the addition of SAS, gabapentin, and memantine to standard chemoradiotherapy with temozolomide or chemoradiotherapy alone improves the outcome of patients with newly diagnosed glioblastoma. Gabapentin inhibits thrombospondin-1 signaling [96, 97] and is approved for the treatment of epilepsy and neuropathic pain. Memantine, the cognitive enhancer approved for the treatment of Alzheimer’s disease, blocks NMDA receptors, thereby inhibiting tumor cell invasion and neuroglial synapse formation [43]. This open-label, randomized, multicenter, phase Ib/II clinical trial is supposed to be completed in December 2026 [98].

Regardless of the clinical trials presented above, studies searching for improved Sxc- inhibitors are currently ongoing [99]. Among them, erastin turned out to be a potent and metabolically stable inhibitor of this transporter that significantly suppressed glioblastoma growth in vivo [100].

Attempts to modulate the level and/or activity of two other Glu transporters impaired in gliomas, namely EAAT1 and EAAT2, are currently limited to preclinical studies. Considering the conflicting reports on the cellular localization of the first one [32, 38], further studies are necessary to determine its role in shaping the glioma phenotype. Regarding EAAT2, current preclinical studies suggest beneficial effects when its expression is restored [33, 81]. Both EAAT2 expression in the brain and its activity resulting in increased Glu uptake are enhanced after treatment with the beta-lactam antibiotic ceftriaxone approved for treating bacterial infections [101, 102]. To the best of our knowledge, the effect of this drug on glioma cells has not been studied so far. Another antibiotic that has been proven to upregulate the expression of EAAT2 is thiamphenicol. Indeed, administration of this drug to rats bearing 9 L gliosarcoma cells significantly increased the level of EAAT2 and the number of neurons in peritumoral tissue, as well as decreased tumor size and extend survival of animals [103]. The results of this study, along with information about the ability of thiamphenicol to penetrate the blood-brain barrier [103], encourage further research on the effectiveness of this compound in glioma models.

### Targeting Glu receptors

The aforementioned evidence that Glu released from glioma cells activates Glu receptors localized on these and neighboring cells, which contributes to the malignant phenotype of glioma, makes these receptors promising therapeutic targets. Talampanel is an oral, noncompetitive antagonist of the AMPA receptors with excellent brain penetration which has been shown to reduce seizures [104]. Hence, its efficacy in patients with recurrent high-grade gliomas was determined in phase II trial (NCT00064363). Unfortunately, even though patients tolerated treatment well and most adverse events were mild and reversible, there was only one partial response among 19 evaluable patients with glioblastoma and no response among 6 patients with anaplastic glioma [105]. Another multicenter phase II trial (NCT00267592) estimated OS for patients with newly diagnosed glioblastoma treated with talampanel during radiotherapy with concurrent and adjuvant TMZ. The results from this study documented encouraging results, with the median survival of patients increased from 14.6 to 20.3 months. Moreover, talampanel was well tolerated and did not increase the toxicity of TMZ [106]. Currently, another AMPA antagonist, perampanel, is subjected in clinical trials. In the first of these trials, antiepileptic efficacy of this drug in patients with glioma will be evaluated (NCT03636958). The second study will evaluate the clinical potential of perampanel to reduce tumor cell network connectivity and tumor growth in patients with progressive glioblastoma [107]. Regarding the antagonists of NMDA receptors, in addition to the aforementioned clinical trial evaluating the effects of memantine in combination with SAS and gabapentin [98], memantine has also been the subject of other clinical trials. The purpose of the first of them (NCT01260467) was to determine the efficiency of this drug in the treatment of recurrent glioblastoma. However, due to poor patient accrual, results are not available. A second ongoing phase I clinical trial (NCT01430351) aims to evaluate the antitumor activity of memantine in combination with TMZ in patients with newly diagnosed glioblastoma. It is worth mentioning that antagonists of both ionotropic and metabotropic Glu receptors inhibited the survival of glioma cells in vitro, but also increased the sensitivity of these cells to cytostatic drugs [73, 108], suggesting that they might be a useful adjuvant treatment to sensitize glioma cells to chemotherapy.

## Conclusions and future perspectives

Studies over the past twenty-five years have indisputably demonstrated a pivotal role of Glu in shaping the phenotype of gliomas. Excessive release of Glu by glioma cells, resulting from impaired transport of this neurotransmitter, causes peritumoral excitotoxic neuronal death, thereby promoting tumor growth. Moreover, it activates Glu receptors, which leads to the development of epileptic seizures. Given the multifaceted role of Glu in the pathobiology of gliomas, pharmacological inhibition of Glu transporters and receptors may be a promising therapeutic strategy for the treatment of these devastating tumors. However, even though the mechanisms underlying the dysregulation of Glu homeostasis in gliomas are relatively well identified, targeting them clinically remains to be a great challenge. This may result from the possible adverse side effects elicited by the anti-glutamatergic drugs on the one hand, and the unique biology of gliomas (i.e. high heterogeneity, plasticity, immunosuppressive tumor microenvironment), which significantly contributes to therapeutic failure, on the other hand. Further studies are needed to design the compounds that target Glu transporters and/or receptors and investigate their efficiency, applied alone or in combination with other drugs, in glioma models.

## Data Availability

Data sharing not applicable to this article as no datasets were generated or analysed during the current study.

## Abbreviations

AEG-1: Astrocyte Elevated Gene-1
AMPA: α-Amino-3-Hydroxy-5-Methyl-4-Isoxazolepropionic Acid
CNQX: Cyanquixaline
CNS: Central Nervous System
D-AP5: D(-)-2-Amino-5-Phosphonopentanoic Acid
EAAT: Excitatory Amino Acid Transporter
FAK: Focal Adhesion Kinase
FDA: Food and Drug Administration
GA: Glutaminase
GLAST: Glutamate/Aspartate-Transporter
Gln: Glutamine
GLT1: Glutamate Transporter 1
Glu: Glutamate
GS: Glutamine Synthase
GSCs: Glioma Stem Cells
GSH: γ-L-Glutamyl-L-Cysteinylglycine
IDH: Isocitrate Dehydrogenase
iGluR: Ionotropic Glutamate Receptors
MAPK: Mitogen-Activated Kinase
mGluR: Metabotropic Glutamate Receptors
NBQX: 2,3-Dihydroxy-6-Nitro-7-Sulfamyl-Benz(f)Quinoxaline
NMDA: N-Methyl-D-Aspartate
PI3K: Phosphatidylinositol-3-Kinase
PPARγ: Peroxisome Proliferator Activated Receptor Gamma
ROS: Reactive Oxygen Species
S-4CPG: (S)-4-Carboxyphenylglycine
SAS: Sulfasalazine
SNAT: Sodium-Coupled Neutral Amino Acid Transporter
Sxc: System x_c_^-^
TME: Tumor Microenvironment
TMZ: Temozolomide
